# Age-Dependent DNA Methylation Variability on the X-Chromosome in Male and Female Twins

**DOI:** 10.3390/epigenomes8040043

**Published:** 2024-11-18

**Authors:** Qihua Tan, Hikmat Alo, Marianne Nygaard, Mette Sørensen, Alisa Saleh, Jonas Mengel-From, Kaare Christensen

**Affiliations:** 1 Epidemiology, Biostatistics and Biodemography, Department of Public Health, University of Southern Denmark, Campusvej 55, DK-5230 Odense, Denmark; hialo17@student.sdu.dk (H.A.); mnygaard@health.sdu.dk (M.N.); msoerensen@health.sdu.dk (M.S.); hi@alisasaleh.com (A.S.); jmengel-from@health.sdu.dk (J.M.-F.); kchristensen@health.sdu.dk (K.C.); 2 Unit of Human Genetics, Department of Clinical Research, University of Southern Denmark, Campusvej 55, DK-5230 Odense, Denmark

**Keywords:** epigenetic instability, X-linked DNA methylation, aging, twins, mortality

## Abstract

We aimed to explore the age-dependent epigenetic variability on the X-chromosome with consideration of X-chromosome inactivation by applying a sex-stratified regression analysis to DNA methylation array data on X-linked CpGs in aging identical twins. We found 13 X-linked CpGs showing age-related significant increase in variability in males (FDR < 0.05) but none in females. In females, we found a significantly higher proportion of CpGs showing increased variability with age among nominally significant (*p* < 0.05) CpGs under inactivation, but not among CpGs escaping inactivation. Survival analysis showed a slight trend of correlation by directional change in the variable CpGs with mortality in males. Compared with females, the male X-chromosome can be more vulnerable to epigenetic instability during aging.

## 1. Introduction

The popularity of genome-wide DNA methylation (DNAm) profiling has enabled many epigenome-wide association studies (EWAS) aimed at exploring the dynamic changes in DNAm during the human aging process, reporting a large number of genomic sites that display age-dependent patterns of differential regulation [1,2,3,4]. Most of the current studies have focused on investigating the directional changes in DNAm levels with age, i.e., CpGs showing increased (hyper-) or decreased (hypo-) methylation patterns [3]. However, these studies have failed to reveal obvious differences in age-related changes in autosomal DNAm between males and females [4,5]. In a meta-analysis performed on four large datasets, Yusipov et al. [6] found that around 11.6% of their analysed CpG sites (38,100 CpGs) displayed sex-dependent methylation patterns, of which 43% showed sex-dependent DNAm changes with age. This may suggest that a large sample size is required to reveal sex differences. Additionally, other studies have demonstrated that males are epigenetically older than females, as indicated by DNA methylation clocks [7,8]. Note that the above conclusions are based on analysing the autosomal chromosomes. In a sex-stratified analysis that considered X-chromosome inactivation (XCI) in females, Li et al. [9] reported sex differences in age-dependent methylation patterns on the X-chromosome. Their study observed an age-dependent increase in X-linked DNAm that is more pronounced in females than in males. These results suggest that the female X-chromosome undergoes more extensive methylation during aging than the male X-chromosome, or simply that the age-dependent X-linked DNAm in males is more variable than in females.

As an alternative to investigating the age-dependent changes in mean levels of DNAm, one can focus on the accumulative epigenetic variability during the aging process, as the loss of control of the epigenome represents an important hallmark of aging [10,11]. Tan et al. [12] analysed the age-dependent variability in DNAm on the autosomes across individuals and found that instability in the DNA methylome during aging is more evident in males than in females, which could impact the sex difference in survival. Likewise, Yusipov et al. [6] reported a much higher age-related increase in methylation variability in males than in females. Consistently, it has been postulated that the variability in DNAm could better reflect individual aberration of the methylome, which can introduce genomic instability that potentially affects lifespan and health span in a sex-dependent manner [13]. 

Inspired by the observed sex difference in age-associated methylation patterns on the X-chromosome [9], this study extends the reported analysis of age-related DNAm variability in the autosomes [12] to the X-chromosome, aiming to explore its role in maintaining epigenomic stability during the aging process. Analysing the age-dependent variability in DNAm on the X-chromosome is technically challenging due to (1) individual heterogeneity in their genetic make-ups and differential environmental exposure—both known to be associated with DNAm [14,15]; and (2) sex differences in copy numbers and X-chromosome inactivation. This study employs a sex-stratified analysis strategy to avoid the complexity of sex differences in the X-chromosome by utilising genome-wide DNAm data collected from 246 monozygotic (MZ) twin pairs in the Danish Twin Registry [16], which controls for individual differences in genetic makeup [14,15,16,17]. Our analytical strategy and study design allow us to model DNAm variability (the intra-pair difference in X-chromosome DNAm patterns) as a function of age between twin pairs (twins in a pair have the same age) in males and females separately. MZ twins in a pair share the same sex and are genetically identical, which enables us to examine the sex- and age-dependent patterns of DNAm variability on the X-chromosome. 

## 2. Materials and Methods

### 2.1. The Samples

The study was conducted using the cohort of Middle Age Danish Twins (MADT) collected by the Danish Twin Registry and included twins born between 1931 and 1952 [18]. Genome-wide DNA methylation data were generated based on whole blood DNA from 492 MZ twin samples (266 males and 226 females) with a mean age of 66 years (age range: 56 to 80 years) using the Infinium Human Methylation 450 K array (Illumina, San Diego, CA, USA) following standard protocols as mentioned elsewhere [19,20]. Date of birth and death data were retrieved from the Danish Civil Registration System in March 2023, at which point 103 twins had died (77 males, 26 females) at a median age of 79.2 years (males: 79.8 years; females: 79.0 years). 

### 2.2. DNAm Data Preprocessing

The raw DNAm measurements were first normalised by the functional normalisation [21] implemented in the *minfi* R-package. For each CpG site, a detection *p* value (a measure of an individual probe’s performance) was calculated for each sample, CpGs with a detection *p* value > 0.01 were treated as missing. CpG sites with more than 5% missing values across the samples were removed from the study. After normalisation and quality control, the DNAm level at each site was summarised as a ‘beta’ value using Illumina’s formula β = M/(M + U + 100), where M and U are methylated (M) and unmethylated (U) signal intensities. For improved statistical properties in subsequent regression analyses, the methylation β values were transformed into M values using the logit transformation, i.e., M = log_2_(β/(1 − β)).

### 2.3. Controlling for Blood Cell Heterogeneity

Our DNAm levels were measured in whole blood, which consists of diverse cell types. Therefore, heterogeneity in cell composition among twin samples can be a confounding factor due to cell-specific DNAm patterns. To control for blood cell heterogeneity, we estimated the proportions of major leukocyte cell types by using the Houseman method [22] implemented in the R-package *minfi* (version 1.52.0). We estimated blood cell proportions in each individual for 6 blood cell types: CD8T, CD4T, natural killer cells, B cells, monocytes, and granulocytes. Before statistical analysis, we adjusted the DNAm level at each site by first regressing the M value on the estimated cell type proportions and then keeping the residual for downstream statistical modelling.

### 2.4. Statistical Modelling of DNAm Variability

The age-dependent DNAm variability is assessed by regressing the square root-transformed intra-pair DNAm difference (absolute value), i.e. |∆DNAm|, on twin pair age as

$$\sqrt{\left|∆DNAm\right|}=\beta_{0}+\beta_{1}age$$

with the null hypothesis *H*_0_: β_1_ = 0. The model detects age-dependent change in DNAm variability if β_1_ is statistically different from 0 using a two-sided test with β_1_ > 0 or β_1_ < 0 suggesting increased or decreased variability in DNAm with increasing age. The above model was fitted to male and female twin pairs separately in our sex-stratified analysis. To correct for multiple testing, we determined the statistical significance of a CpG by calculating the false discovery rate (FDR) [23], with FDR < 0.05 defined as significant. 

### 2.5. Survival Analysis

To examine the impact of age-dependent variability in DNAm on mortality, we further assessed, for each CpG on the X-chromosome, the association with risk of death using the survival information retrieved from national death records. The Cox proportional hazards model was fitted with DNAm as an explanatory variable and twin pairing as clusters to control for intra-pair correlation while including age at blood sampling for adjustment,

$$H\left(t\right)=H_{0}\left(t\right)\exp\left(b_{1}DNAm+b_{2}age_{blood}\right).$$

Here, *H*_0_(*t*) is the baseline hazard at age t, which is the hazard of an individual with the predictors set to zero, and b_1_ and b_2_ are the regression coefficients.

## 3. Results

**Figure 1:**
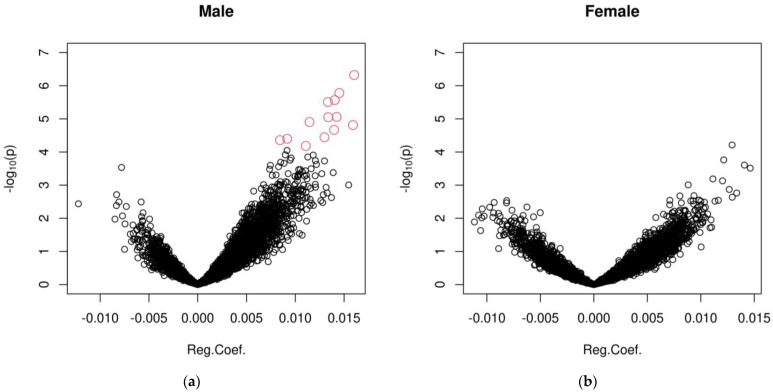
Volcano plot of association test on DNAm variability in male (**a**) and female (**b**) twins, with negative log of the *p* value (base 10) for each CpG site on the Y-axis, and age-dependent change in DNAm variability (as regression coefficient for age) on the X-axis. The red dots represent CpGs showing significant age-related intra-pair methylation discordance with FDR < 0.05.

In the sample of middle-aged monozygotic twin pairs, the gender ratio was 1.18 (266 males and 226 females), and a total of 10179 X-linked CpGs were available for statistical testing after data preprocessing (CpGs homologous on the X and Y chromosomes were removed). Among these CpGs, 13 CpGs were identified as having significant age-dependent variability with a FDR < 0.05 (corresponding to *p* < 6.59 × 10^−5^) in males (Table 1, Supplementary Table S1). No CpGs were found to be significant in females after adjusting for multiple testing (Supplementary Table S2). In the volcano plot in Figure 1, all 13 significant CpGs display increased variability with increasing age in males. Note that Figure 1 also reveals a predominant pattern of increasing variability in most of the CpG sites in males, whereas the trend is much less pronounced in females. The Manhattan plots (Supplementary Figures S1 and S2) show that the significant CpGs in males are evenly distributed along the X-chromosome. 

**Figure 2:**
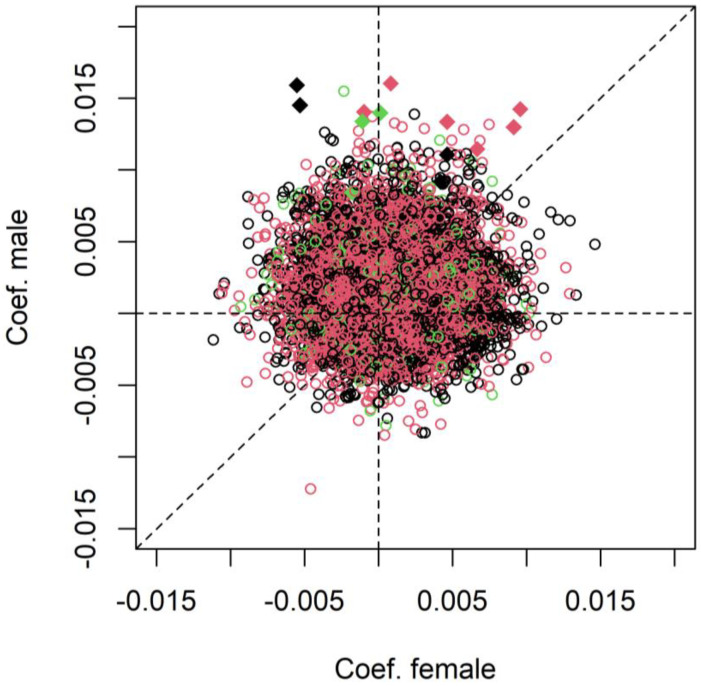
A scatter plot showing the estimated effect size of age (regression coefficient) on DNAm variability in males on the Y-axis plotted against that in females on the X-axis. The red and green dots represent CpGs detected as under and escaping XCI in females, respectively. The diamond represents significantly variable CpGs in males.

We further investigated whether X-chromosome inactivation (XCI) specifically influences age-related DNAm stability in females by plotting the regression coefficient of age in males against that in females, with CpGs under XCI marked in red and those escaping XCI marked in green (Figure 2). XCI status was determined in our previous study on XCI by Li et al. [9]. CpGs under or escaping XCI in females are predominantly more variable with increasing age in males, a pattern that applies to all X-linked CpGs on the male X-chromosome. The thirteen significantly variable CpGs in males all are more variable with increasing age, including five CpGs under XCI and three CpGs escaping XCI in females. Although no significantly variable CpGs were found in females, a total of 141 CpGs under XCI showed nominal significance with *p* < 0.05. Among them, 101 CpGs gained variability with aging (71.63%). A binomial test assuming an equal number of increased and decreased variability (i.e., 50%) showed a value of *p* = 2.88e-07, indicating a predominant pattern of increased variability in XCI CpGs of nominal significance. A similar test on twenty-five CpGs that escape XCI, with a nominal significance of *p* < 0.05 for age-related DNAm variability (sixteen CpGs with increased and nine CpGs with decreased variability), showed a *p* value of 0.23, suggesting no difference in the up and down patterns of DNAm variability among CpGs escaping XCI. 

**Figure 3:**
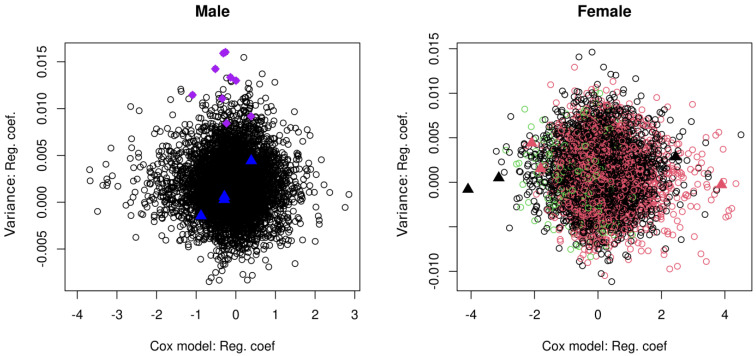
Scatter plots demonstrating the relationship between age-related DNAm variability (Y-axis, regression coefficient for age) and mortality (X-axis, Cox regression coefficient) in males (**left**) and females (**right**). The purple diamonds in the left panel represent CpGs significant for DNAm variability in males. The blue triangles in the left panel show CpGs significant for risk of death in males. The large triangles in the right panel are the 6 CpGs significant for mortality in females. CpGs under and escaping XCI in females are coloured red and green respectively in the right panel. The rest of X-linked CpGs are coloured black.

Next, we explored the relationship between DNAm variability and an individual’s risk of death, as well as the role of XCI. We first fitted Cox proportional hazards models to the DNAm data for males and females, controlling for age at blood sampling. After correction for multiple testing, only four CpGs in males and nine CpGs in females were found to be significant (FDR < 0.05) (Supplementary Figure S3, Supplementary Tables S3 and S4). Figure 3 presents the relationship between DNAm variability and mortality by plotting the coefficient for variability against the Cox model coefficient for DNAm in males and females. In males, the thirteen significantly variable CpGs (purple diamonds) were not involved in survival and the four CpGs significantly associated with mortality (blue triangles) did not display high age-dependent variability. In the female data plot, CpGs under XCI are represented in red (3162 CpGs) and CpGs escaping XCI in green (424 CpGs). The nine CpGs significantly associated with mortality (triangles) are all distributed around zero on the Y-axis, indicating low variability. Note that in the plot for females in Figure 3, the CpGs under XCI (red dots) are more shifted to the right side of the plot, indicating an increased risk of death with Cox regression coefficient > 0 (1658 out of 3162 CpGs, 52.44%; binomial test *p* < 6.5 × 10^−^³ with null hypothesis of 50%). CpGs escaping XCI (green dots) are primarily located to the left, which corresponds to reduced risk of death with Cox regression coefficient < 0 (302 out of 424 CpGs, 71.23%; binomial test *p* < 1 × 10^−22^ with null hypothesis of 50%), a pattern that is independent of DNAm variability.

**Figure 4:**
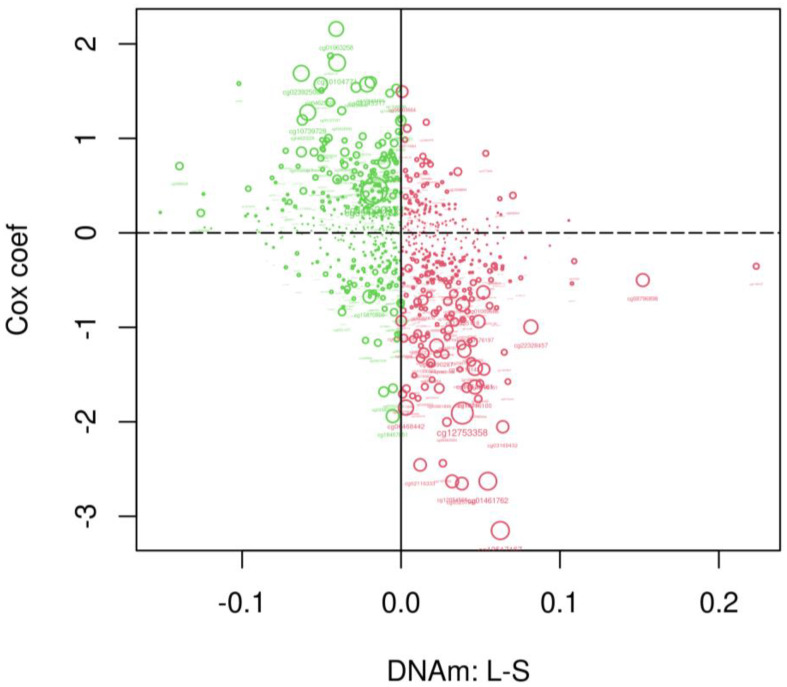
Scatter plot showing the Cox regression coefficient (the effect size on mortality) of each varying CpG (Y-axis) plotted against its mean of intra-pair DNAm difference (X-axis, long-lived twin (L) minus short-lived twin (S)) with CpGs having L−S > 0 coloured red and CpGs having L−S < 0 coloured green. The bigger the size of the dots, the more statistically significant in the survival analysis.

The relationship between mortality and CpG methylation variability examined above does not consider the directional effect of intra-pair DNAm discordance, specifically with twins discordant for survival (40 twin pairs with 1 twin deceased and 1 twin alive; 15 twin pairs in which both twins died). Since we only detected CpGs showing significant variability in males, we further focused on the CpGs showing nominally significant variability in males (*p* < 0.05, 978 CpGs) to investigate whether DNAm discordance between the long- and short-lived co-twins of each twin pair is associated with the risk of death estimated from the Cox regression analysis above. Figure 4 displays the results by plotting the Cox regression coefficient (the effect size from the mortality analysis) for each varying CpG (Y-axis) against its mean intra-pair DNAm difference (long-lived twin minus short-lived twin). It can be seen that CpGs favouring survival (negative Cox regression coefficients) with small *p* values (large dots) tend to be more methylated in the long-lived twins (L−S > 0), while CpGs increasing the risk of death (positive Cox regression coefficients) with small *p* values tend to be more methylated in the short-lived twins (L−S < 0). 

## 4. Discussion

We have previously analysed the age-dependent DNAm variability in the autosomes of MZ twins and reported striking sex differences with significantly increased variability in males but not in females [12]. In the present study, we extended the variability analysis to the X-chromosome and meanwhile explored the association of XCI in females with X-chromosome epigenetic stability. Our analysis revealed a similar pattern of sex differences in X-linked DNAm variability as seen in the autosomes and found that the X-linked DNAm variability in females is independent of XCI. Combining our results on the autosomes and the X-chromosome, it is evident that the DNA methylome in males is more vulnerable in terms of epigenetic stability than in females. Importantly, in our analysis, DNAm variability is measured as the intra-pair differences in MZ twin pairs, meaning that the genetic regulation over DNAm (e.g., by methylation quantitative trait loci (mQTLs)) is controlled for, as it is partly cancelled out by the MZ twin design. As a result, the pattern of sex differences in DNAm variability observed here is likely to be due to differential environmental exposures by the two sexes. Based on epidemiological and clinical studies, Silveyra et al. [24] suggested that males and females respond differently to ozone and PM exposure, indicating sex differences in susceptibility to environmental exposures. Unfortunately, molecular studies aiming to elucidate sex-specific mechanisms have been rare. Our finding of sex-dependent differential variability in the DNA methylome offers an epigenetic explanation for the environmentally induced differential regulation of our genome, which can help with interpreting the observed sex differences in health traits in population-based epidemiological studies and clinical investigations. 

A recent analysis of X-chromosome DNA methylation variability with aging using unrelated individuals reported age-dependent widespread epigenetic variability on the inactive X-chromosome [25]. Though we found no significantly variable X-linked CpGs in females, the distribution of a significantly high proportion of CpGs with increasing age-related variability at nominal significance (*p* < 0.05) among CpGs under XCI, but not among CpGs escaping XCI, seems to comply with the results of Liu et al. [25]. However, the insignificant difference in the proportions of up and down patterns in CpGs escaping XCI could also be due to the small number of them as compared with CpGs under XCI. As an important biological phenomenon of evolutionary origins, XCI is contributed by both genetic and epigenetic mechanisms [26]. Additionally, the functionality of Xist, the major effector of the X-inactivation process, is also shown to be influenced by both genetic and epigenetic variations [27]. Unlike our twin model, which controls for individual genetic make-ups and age in calculating intra-pair DNAm variations, the use of unrelated individuals leaves all relevant genetic and epigenetic (mQTL) variations unadjusted, which could potentially increase the observed DNAm variability. Since XCI is unique to females, ignoring the multiple factors involved in the XCI process makes the conclusions on age-related DNAm variability incomparable between males and females. This further illustrates the usefulness of twins in studying the sex difference in X-linked DNAm variability in human aging. 

As shown in Figure 3, the CpGs with a high association (i.e., effect size indicated by Cox regression coefficients) with survival tend to be distributed in regions of low variability both in males and in females. Note that the cohort used in this study is relatively young, with many individuals still alive. Among the 266 male samples, only 77 deaths (29%) were observed. The high censoring rate could have reduced the effectiveness of our statistical analysis in testing the effects of highly varying CpGs on survival. The scatter plot for females in Figure 3 also reveals an important phenomenon regarding XCI status and survival. The pattern shows that increased methylation at the CpGs escaping XCI could reduce the hazard of death in females, as these CpGs (green empty dots) tend to have negative regression coefficients in their Cox models. However, as revealed by Li et al. [9], CpGs escaping XCI are overwhelmingly demethylated with increasing age, and the clear shift by CpGs under (red) or escaping (green) XCI could indicate a potential risk during female aging conferred by genes escaping XCI. Posynick and Brown [28] discussed the evolutionary perspective of escaping XCI and the important contribution of escape genes to multiple diseases in mouse models. In humans, many diseases are related to XCI escape due to the overexpression of affected genes in female cells, including autoimmune diseases (systemic lupus erythematosus and autoimmune thyroid diseases) and some psychiatric disorders (bipolar disorder and major depression) [29]. Our results highlight the importance of studying escape genes vs. inactivated genes in relation to their association with sex-dependent diseases and mortality. 

Although Figure 3 does not reveal a relationship between DNAm variability and survival, our further examination based on direction of intra-pair DNAm discordance between a limited number of longer- and shorter-lived co-twins (Figure 4) showed a slight trend of age-related change in X-linked DNAm that is in favor of survival. Future updates to the mortality data of the cohort will enable more accurate and efficient analysis to elucidate the relationship between age-dependent DNAm variability and mortality. 

## 5. Conclusions

Our twin-based analysis of DNAm variability on the X-chromosome identified significantly variable CpG sites with increasing age in males, but no significant sites were found in females. Our results suggest that the male X-chromosome can be more vulnerable to epigenetic instability during the aging process when compared to females. The observed sex difference in age-related DNAm variability could potentially impact survival in a sex-dependent manner. Independent studies are required to replicate and validate our findings. 

## Figures and Tables

**Table 1:** The 13 significant CpGs detected in males.

CpGs	Reg. Coef.	t Value	*p* Value	FDR	Position (Base Pairs)	UCSC Ref Gene Name	UCSC Ref Gene Group
cg06936709	0.0160	5.3001	4.77 × 10^−7^	0.004	83757476	HDX	TSS200
cg12972869	0.0145	5.019	1.66 × 10^−6^	0.006	152711957	TREX2	TSS200
cg15048430	0.0140	4.9057	2.71 × 10^−6^	0.006	54384161	WNK3	1stExon; 5′UTR
cg01309671	0.0133	4.8742	3.10 × 10^−6^	0.006	133941225	FAM122C	5′UTR; 1stExon; Body
cg26179626	0.0142	4.6285	8.75 × 10^−6^	0.012	139015804		
cg26816294	0.0134	4.6242	8.91 × 10^−6^	0.012	15693836	CA5BP	Body
cg00743068	0.0115	4.5424	1.25 × 10^−5^	0.014	77041832	ATRX	TSS200
cg03244736	0.0159	4.4909	1.54 × 10^−5^	0.015	114252923	IL13RA2	TSS1500
cg22268449	0.0140	4.4084	2.15 × 10^−5^	0.019	47053156	UBA1	TSS200; 5′UTR
cg24547622	0.0130	4.2795	3.59 × 10^−5^	0.029	23761653	ACOT9	TSS1500
cg01645836	0.0092	4.2519	4.00 × 10^−5^	0.029	36081090	CXorf59	5′UTR
cg13274727	0.0084	4.2286	4.38 × 10^−5^	0.029	15808320	ZRSR2	TSS1500
cg15737490	0.0111	4.1232	6.59 × 10^−5^	0.04	70390672	NLGN3	3′UTR

## Data Availability

The authors certify that this manuscript reports original X-linked DNA methylation data. According to current Danish and EU legislations, transfer and sharing of individual-level data require prior approval from the Danish Data Protection Agency. Our present local data protection rules do not allow individual-level data to be shared in public databases. For these reasons, the raw data cannot be deposited in a public database. However, we welcome any individual request for data sharing by directing requests directly to Qihua Tan at qtan@health.sdu.dk. R codes used for data analysis are available upon request by directly contacting the corresponding author at qtan@health.sdu.dk.

## Supplementary Materials

The following supporting information can be downloaded at: https://www.mdpi.com/article/10.3390/epigenomes8040043/s1, Figure S1. Manhattan plots for site-specific significance (negative log of the *p* value with base 10) on DNAm variability for male twins. Figure S2. Manhattan plots for site-specific significance (negative log of the *p* value with base 10) on DNAm variability for female twins. Figure S3. Volcano plots for results of survival analysis in male (a) and female (b) twins, with negative log of the *p* value (base 10) for each CpG site on the y axis. The red dots represent significant CpGs with FDR < 0.05. Table S1. Annotated age-dependent DNAm variability association results for males. Table S2. Annotated age-dependent DNAm variability association results for females. Table S3. Cox regression results for X-linked CpGs in males. Table S4. Cox regression results for X-linked CpGs in females.
