# Supplementary figures and images for: Age-Dependent DNA Methylation Variability on the X-Chromosome in Male and Female Twins
Source: Epigenomes. 2024 Nov 18;8(4):43. doi: 10.3390/epigenomes8040043 (PMC11586961; doi:10.3390/epigenomes8040043)

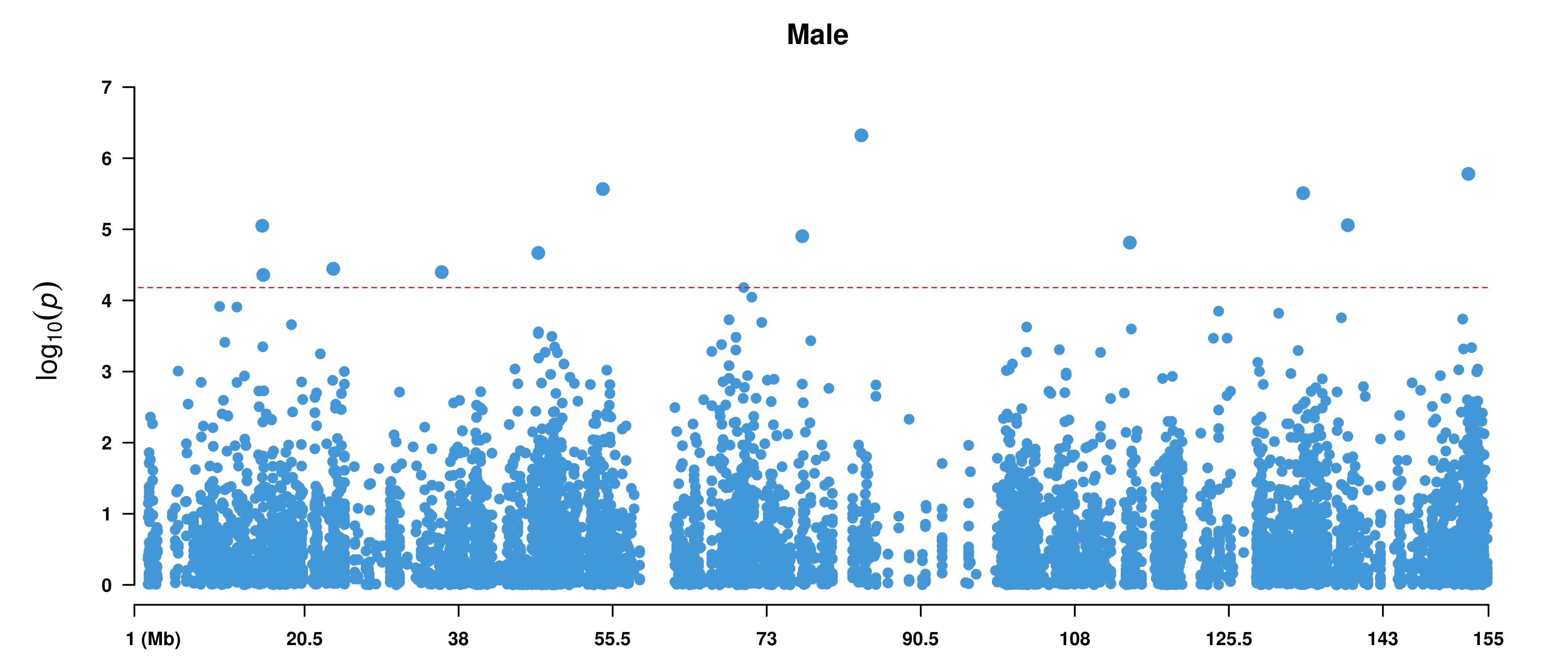

Supplement: Supplementary file 1 [file epigenomes-08-00043-s001.zip › Supplementary Figure S1_Manhattan_m.jpg]

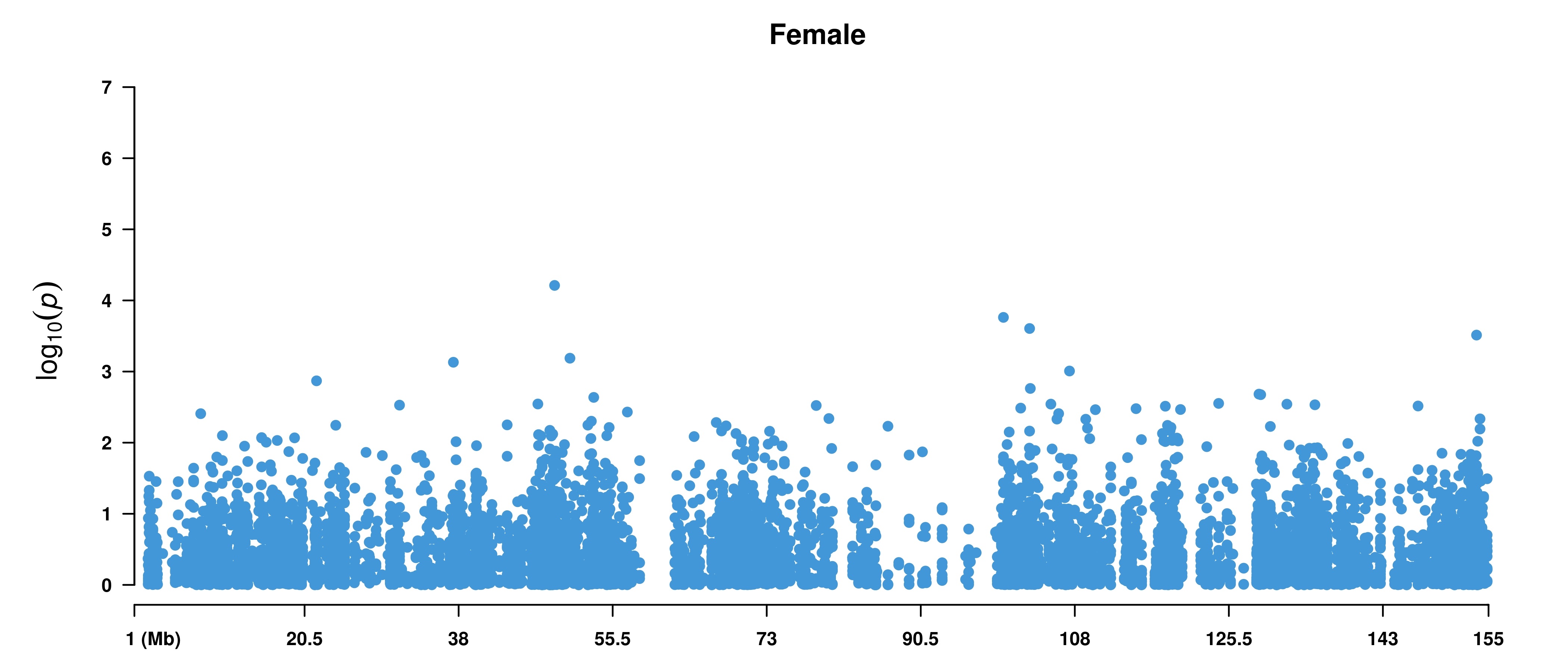

Supplement: Supplementary file 1 [file epigenomes-08-00043-s001.zip › Supplementary Figure S2_Manhattan_f.jpg]

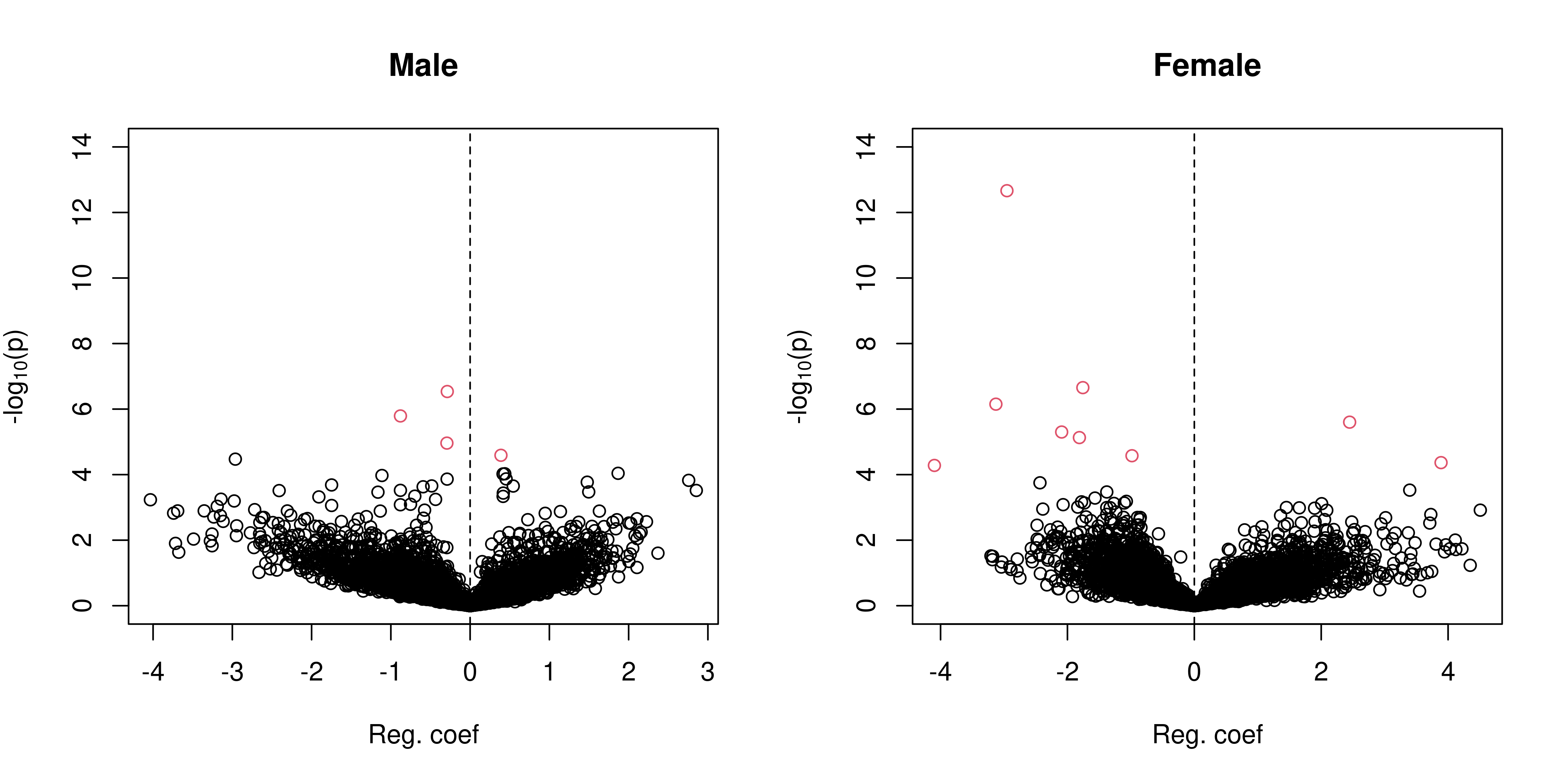

Supplement: Supplementary file 1 [file epigenomes-08-00043-s001.zip › Supplementary Figure S3_VolcanoPlot_Cox.png]
